# Factors Associated with Risk of Diabetic Complications in Novel Cluster-Based Diabetes Subgroups: A Japanese Retrospective Cohort Study

**DOI:** 10.3390/jcm9072083

**Published:** 2020-07-02

**Authors:** Hayato Tanabe, Haruka Saito, Akihiro Kudo, Noritaka Machii, Hiroyuki Hirai, Gulinu Maimaituxun, Kenichi Tanaka, Hiroaki Masuzaki, Tsuyoshi Watanabe, Koichi Asahi, Junichiro Kazama, Michio Shimabukuro

**Affiliations:** 1 Department of Diabetes, Endocrinology and Metabolism, Fukushima Medical University, Fukushima 960-1295, Japan; htanabe@fmu.ac.jp (H.T.); saito-h@fmu.ac.jp (H.S.); a-kudoh@fmu.ac.jp (A.K.); noritaka@fmu.ac.jp (N.M.); hiroyuki@fmu.ac.jp (H.H.); gulinumaimaituxun@gmail.com (G.M.); 2 Department of Internal Medicine, Ohara General Hospital, Fukushima 960-8611, Japan; 3 Department of Internal Medicine, Shirakawa Kosei General Hospital, Fukushima 961-0005, Japan; 4 Department of Nephrology and Hypertension, Fukushima Medical University, Fukushima 960-1295, Japan; kennichi@fmu.ac.jp (K.T.); jjkaz@fmu.ac.jp (J.K.); 5 Division of Endocrinology, Diabetes and Metabolism, Hematology, Rheumatology (Second Department of Internal Medicine), University of the Ryukyu, Okinawa 903-0215, Japan; hiroaki@med.u-ryukyu.ac.jp; 6 Department of General Medicine, Tokyo-Kita Medical Center, Tokyo 115-0053, Japan; twat0423@fmu.ac.jp; 7 Department of Nephrology and Hypertension, Iwate Medical University, Iwate 020-8505, Japan; asahik@iwate-med.ac.jp

**Keywords:** type 2 diabetes, homeostasis model assessment (HOMA), chronic kidney disease, retinopathy, coronary artery disease

## Abstract

Diabetes is a complex and heterogeneous disease, making the prediction of the risks of diabetic complications challenging. Novel adult-onset diabetes subgroups have been studied using cluster analysis, but its application in East Asians remains unclear. We conducted a retrospective cohort study to elucidate the clinical utility of cluster-based subgroup analysis in the Japanese population. Cluster analysis based on anti-glutamate decarboxylase antibody (GAD antibody) levels, age at diagnosis, body mass index (BMI), hemoglobin A1c (A1c), and homeostatic model assessment 2 estimates of β-cell function and insulin resistance was performed in 1520 diabetic patients. The risk of developing diabetic complications was analyzed using Kaplan–Meier analysis and the Cox proportional hazards model. By cluster analysis, we identified five distinct subgroups of adult-onset diabetes in the Japanese population. The risk of diabetic complications varied greatly among the clusters. Patients with severe autoimmune diabetes or severe insulin deficiency diabetes were at an increased risk of diabetic retinopathy, and those with severe insulin resistant diabetes (SIRD) had the highest risk of developing diabetic kidney disease (DKD). After adjusting for uncorrectable and correctable risk factors, SIRD was found to be an independent risk factor for DKD. In conclusion, we identified five subgroups of adult-onset diabetes and the risk factors for diabetic complications in the Japanese population. This new classification system can be effective in predicting the risk of diabetic complications and for providing optimal treatment.

## 1. Introduction

In current clinical practice, diabetes mellitus is mainly classified as type 1 diabetes or type 2 diabetes [1]. In particular, type 2 diabetes is a complex, heterogeneous disease that has multiple variables, such as age of onset, clinical characteristics, and glycemic control [2,3]. Therefore, classifications of type 2 diabetes that predict the risk of complications and recommend appropriate treatment have been actively studied [4,5,6,7].

Ahlqvist and colleagues recently proposed an interesting novel classification system for diabetes [8]. They performed data-driven cluster analysis using six variables (glutamate decarboxylase antibody (GADA) levels, age at diagnosis, body mass index (BMI), hemoglobin A1c (A1c), and homeostasis model assessment estimates of beta-cell function (HOMA2-B) and insulin resistance (HOMA2-IR)) at the onset of diabetes in a Scandinavian cohort and found five exclusive diabetes subgroups (SAID: severe autoimmune diabetes, SIDD: severe insulin-deficient diabetes, SIRD: severe insulin-resistant diabetes, MOD: mild obesity-related diabetes, MARD: mild age-related diabetes). They reported a remarkable difference in cumulative incidence of diabetic complications among the five subgroups. Reports on the American, Chinese [9], German [10], and UK [11] populations have revealed a global reproducibility of the complication trajectories.

Since East Asians, including Japanese, develop diabetes with relatively low adiposity and insulin resistance as compared to those in Caucasians [12,13,14], it is suggested that impaired insulin secretion is more involved in the onset of diabetes in this population. These findings suggest that the distribution of diabetes subgroups and its impact on diabetic complications may differ between non-Caucasians, such as Japanese, and Caucasians. However, the issue remains to be elucidated.

Therefore, we conducted a retrospective cohort study to identify the cluster-based diabetes subgroups of adult-onset diabetes in the Japanese population and their impact on the prediction of diabetic complications.

## 2. Materials and Methods

### 2.1. Study Design and Population

This is an observational retrospective study that examined a total of 1520 people, including 917 of 2724 diabetic patients enrolled in the Fukushima chronic kidney disease (CKD) cohort and 603 diabetic patients enrolled in the Fukushima Diabetes, Endocrinology, and Metabolism (DEM) cohort (Figure S1). The Fukushima CKD cohort study is a survey to investigate the characteristics and outcomes, such as cardiovascular events, end-stage kidney disease, and death, of patients with pre-CKD or CKD in Fukushima Prefecture, Japan [15]. The Fukushima DEM cohort study is a survey of pre-diabetic and diabetic patients at Fukushima Medical University for clarifying risk factors for the onset and progression of diabetes and its complications. Both study protocols were approved by the Fukushima Medical University Ethics Committee (the Fukushima CKD cohort #1456, the Fukushima DEM cohort #29118). Written informed consent was received from patients in the CKD cohort between October 2012 and September 2014 and from patients in the Fukushima DEM cohort between January 2018 and December 2019. Data from all individuals were checked to avoid duplication in the two cohorts.

Their first visit to hospital for treatment or evaluation of diabetes mellitus in the medical records was considered as baseline. Between January 2003 and March 2017 in the Fukushima CKD cohort and between January 2003 and November 2019 in the Fukushima DEM cohort, all medical information (such as history of diabetes, past events and lifestyle, family history, prescription drug use, blood and urine test results, and registered International Classification of Disease (ICD-10) codes) was collected from electrical medical records and/or paper medical charts by trained medical staff and doctors. Diabetes was defined by ICD-10 codes E10–14 or the following diagnostic criteria: fasting plasma glucose ≥126 mg/dL, random plasma glucose ≥200 mg/dL but in a patient with classic symptoms of hyperglycemia or hyperglycemic crisis, or A1c ≥6.5% (48 mmol/mol). Among the 3444 patients surveyed, 1520 were diagnosed as having diabetes (Table S1). In addition, patients with secondary diabetes (such as pancreatic diabetes, drug-induced diabetes, monogenic diabetes; *n* = 20); patients with diabetes onset before age 18 years (*n* = 36); patients with missing data, such as BMI and serum C-peptide or insulin level (*n* = 197); and extreme outliers (>5 SDs from the mean; *n* = 12) were excluded as in Ahlqvist et al. [8] Five HOMA2-B >5 SD (no C-peptide provided) or seven HOMA2-IR >5 SD included marked hyperinsulinemia with a range between fasting immunoreactive insulin (IRI) 30.8–50.5 μU/mL. In the diabetic patients in second cohort study, 315 of 1520 patients with diabetes mellitus (20.7%) were checked for GADA. We regarded the patients who had not been checked for GADA as GADA negative. Among 1255 patients in the full analysis set, 785 (51.6%) for serum C-peptide, 555 (36.5%) for insulin, and 85 for C-peptide and insulin were checked. Patients (*n* = 180) without insulin nor C-peptide were excluded (*n* = 180) (Figure S1). In the patients checked with both C-peptide and insulin, C-peptide was calculated for HOMA2-B and HOMA2-IR. Finally, 1255 diabetic patients were included in the study. Furthermore, those who were diagnosed with non-diabetic kidney disease, such as chronic glomerulonephritis, vasculitis, polycystic kidney disease, and renal cancer, were excluded from the analysis for diabetic kidney disease.

### 2.2. Blood Measurements

HOMA2-B and HOMA2-IR were calculated with the HOMA calculator based on fasting plasma glucose and fasting serum C-peptide concentrations measured at the baseline or the time point closest to the baseline [16]. In cases in which serum C-peptide levels were not measured, the HOMA2 index was calculated using plasma insulin concentrations. C-peptide levels were measured for patients on insulin therapy. GADA positivity was measured using ELISA (cutoff < 5.0 U/mL) or a radioimmunoassay (cutoff <1.5 U/mL). We calculated the estimated glomerular filtration rate (eGFR) using the Japanese formula for GFR estimation, i.e., eGFR (mL/min/1.73 m^2^) = 194 × serum creatinine (mg/dL)^−1.094^ × age (years)^−0.287^ [17].

### 2.3. Definition of Diabetes Subgroups and Diabetic Complications

Type 1 diabetes was defined as having GADA positivity and a C-peptide level < 0.3 nmol/L. Adult latent autoimmune diabetes (LADA) was defined as having GADA positivity and a C-peptide level ≥0.3 nmol/L. The definition of diabetic kidney disease (DKD) was having chronic kidney disease (CKD) and/or proteinuria. CKD was defined as having eGFR <60 mL/min/1.73 m^2^ lasting more than 90 days. Proteinuria was defined as a reading of 1 + on dipstick urine tests lasting more than 90 days. End-stage kidney disease was defined as having an eGFR <15 mL/min/1.73 m^2^ or receiving renal replacement therapy. Diabetic retinopathy was diagnosed by an ophthalmologist on the basis of fundus examination or defined on the basis of ICD-10 codes E103, E113, or E143. Diabetic polyneuropathy was defined as meeting the diagnostic criteria [18] or by ICD-10 codes E104 or E114. Coronary artery disease was defined by ICD-10 codes I20–21, I24, I251, or I253–259. Stroke was defined by ICD-10 codes I60–61 or I63–64. Peripheral artery disease was defined by ICD-10 code I739. Hypertension was defined as systolic blood pressure ≥140 mmHg, diastolic blood pressure ≥90 mmHg, or administration of antihypertensive drugs. Dyslipidemia was defined as total cholesterol ≥220 mg/dL, triglyceride ≥150 mg/dL, high density lipoprotein (HDL) cholesterol <40 mg/dL, low density lipoprotein (LDL) cholesterol ≥140 mg/dL, or administration of lipid-lowering drugs. In the Japanese guideline for dyslipidemia, the definition of dyslipidemia are triglyceride ≥150 mg/dL, HDL cholesterol <40 mg/dL, and LDL cholesterol ≥140 mg/dL and the targets of lipid treatment in diabetic patients are triglyceride <150 mg/dL, HDL cholesterol ≥40 mg/dL, and LDL cholesterol <120 mg/dL [19]. Nonalcoholic fatty liver disease (NAFLD) was defined as being a non-drinker and having a hepatic steatosis index (calculated from aspartate transaminase (AST), alanine aminotransferase (ALT), and BMI) >36 [20].

### 2.4. Cluster Analysis

On the basis of the report by Ahlqvist and colleagues [8], we applied the TwoStep clustering method in the 1255 diabetic participants. For the first step, we estimated the appropriate number of clusters by the silhouette width method. We then performed a hierarchical cluster analysis as the second step. Hierarchical clustering was carried out for 2 to 15 clusters using log-likelihood as a distance measure and Schwarz′s Bayesian criterion for clustering. Only for GADA-negative individuals, was k-means clustering performed with a k value of 4 (number of runs 100). All GADA-positive individuals were manually assigned to a separate cluster because k-means clustering does not incorporate binary variables.

### 2.5. Statistical Analysis

Continuous values are expressed as median (first quartile-third quartile) because they are nonparametric data. Categorical variables are shown as percentages and were analyzed using the Kruskal–Wallis test and the post hoc Holm test. Univariate survival analysis was carried out using the Kaplan–Meier method and the results were analyzed using a log rank test. In a multivariate analysis using the Cox proportional hazards model, hazard ratios (HR) were calculated for model 1, 3, and 5, with non-modifiable factors (age, sex, duration of diabetes), and model 2, 4, 6, with modifiable risk factors. The HR was compared to that of the MARD cluster, which is the most frequent cluster, as a reference. *p*-Values < 0.05 were considered as statistically significant. Statistical analyses were carried out using SPSS version 26 (SPSS, Inc., Chicago, IL, USA). A multiple log rank test was carried out using survival package in R version 3.6.3. (R Foundation, Vienna, Austria).

## 3. Results

### 3.1. Cluster Distribution and Characteristics at Baseline

**Figure 1:**
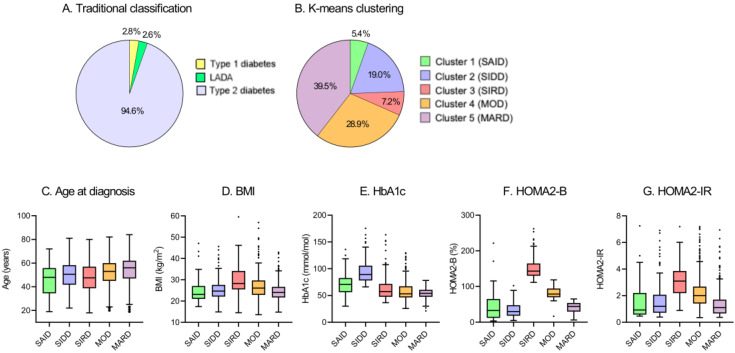
Distribution and characteristics of studied patients. (**A**) Distribution of studied patients according to traditional classification. (**B**) Distribution of study patients according to k-means clustering. Characteristics of (**C**) age at diagnosis, (**D**) body mass index (BMI), (**E**) hemoglobin A1c (HbA1c), (**F**) homeostatic model assessment 2 estimates of β-cell function (HOMA2-B), and (**G**) homeostatic model assessment 2 estimates of insulin resistance (HOMA2-IR) for each cluster. SAID: severe autoimmune diabetes; SIDD: severe insulin-deficient diabetes; SIRD: severe insulin-resistant diabetes; MOD: mild obesity-related diabetes; MARD: mild age-related diabetes; LADA: adult latent autoimmune diabetes.

Using the TwoStep cluster analysis, five subgroups were identified (Table 1). Alternatively, k-means clustering performed in GADA-negative patients also showed a similar distribution to that observed in the TwoStep method. Of the 1255 diabetic patients, 35 (2.8%) had type 1 diabetes, 33 (2.6%) had LADA, and 1187 (94.6%) had type 2 diabetes (Figure 1A). The frequency and distribution of the five clusters obtained by k-means clustering are shown in Table 1 and Figure 1B, respectively. The five subgroups were similar in characteristics (Table 1, Figure 1B–G, and Table S1) to the subgroups in the study by Ahlqvist et al., and so we gave the five subgroups the same class names (cluster 1: severe autoimmune diabetes (SAID), cluster 2: severe insulin-deficient diabetes (SIDD), cluster 3: severe insulin-resistant diabetes (SIRD), cluster 4: mild obesity-related diabetes (MOD), cluster 5: mild age-related diabetes (MARD)).

Cluster 1 (SAID), including 68 (5.4%) patients, had lower ages at diagnosis and higher A1c levels as compared with reference cluster 5 (MARD, Table 1 and Table S1). Cluster 2 (SIDD), including 238 (19.0%) patients, had low HOMA2-B and higher A1c levels. Cluster 3 (SIRD), including 90 (7.2%) patients, was characterized by young age, a high BMI, and a high HOMA2-IR. Cluster 4 (MOD), including 363 (28.9%) patients, had a slightly younger age, a high BMI, a high HOMA2-B, and a high HOMA2-IR, with comparable A1c levels. Cluster 5 (MARD), including 496 (39.5%) patients, was the most common cluster and had the oldest patients among the five subgroups.

### 3.2. Survival Analysis for the Development of Diabetic Complications

**Figure 2:**
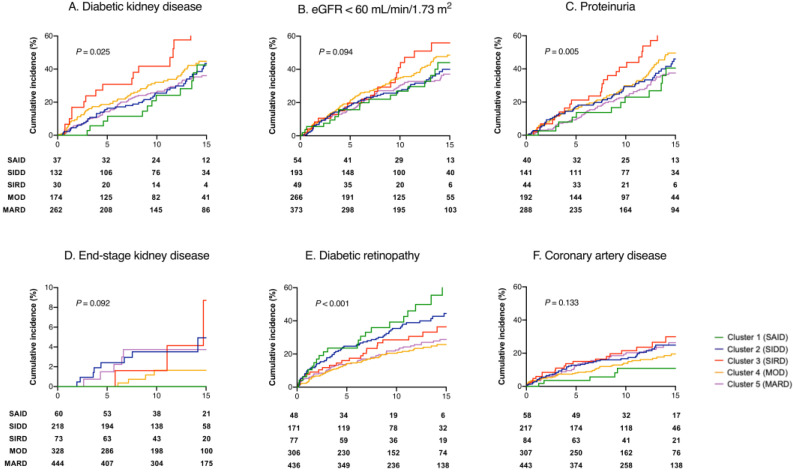
Progression of diabetic complication between subgroups. Kaplan–Meier curves for the development of (**A**) diabetic kidney disease (DKD: eGFR < 60 mL/min/1.73 m^2^ or proteinuria), (**B**) eGFR < 60 mL/min/1.73 m^2^, (**C**) proteinuria, (**D**) end-stage kidney disease, (**E**) diabetic retinopathy, and (**F**) coronary artery disease in patients between SAID (green lines), SIDD (blue lines), SIRD (red lines), MOD (orange lines), and MARD (purple lines). SAID: severe autoimmune diabetes; SIDD: severe insulin-deficient diabetes; SIRD: severe insulin-resistant diabetes; MOD: mild obesity-related diabetes; MARD: mild age-related diabetes; eGFR: estimated glomerular filtration rate.

The results of the Kaplan–Meier survival analyses are depicted in Figure 2 and Table S2. The incidence of DKD was higher in the SIRD subgroup (HR 1.71, 95% CI 1.14–2.55, multiple Log rank *p* = 0.009), and the incidence of both CKD (HR 1.60, 1.03–2.47, *p* = 0.035) and proteinuria (HR 2.19, 1.43–3.36, *p* < 0.001) were higher in the SIRD subgroup as well. Diabetic retinopathy was frequent in the SAID (HR 2.35, 1.49–3.73, *p* < 0.001) and SIDD subgroups (HR 1.78, 1.30–2.43, *p* < 0.001). There was no significant difference in coronary artery disease incidence (*p* = 0.132).

The results of multivariate analysis of DKD, diabetic retinopathy, and coronary artery disease are shown in Table 2. According to model 1, the SIRD and MOD clusters were at a higher risk of DKD than was the MARD cluster. In model 2, even after adjusting for modifiable risk factors of DKD, the SIRD cluster remained at a high risk for DKD. As shown in Table 2, the SAID and SIDD subgroups were at a high risk for diabetic retinopathy in model 3; in model 4, only the SAID cluster showed a significant risk for diabetic retinopathy, as well as having increased baseline diabetes duration and A1c. As shown in Table 2, the SIDD and SIRD clusters were at a high risk of coronary artery disease in model 5, but when adjusted for modifiable factors, such as smoking, hypertension, and dyslipidemia, neither of the two clusters was at risk of coronary artery disease in model 6.

## 4. Discussion

We conducted a retrospective cohort study for clarifying clustering-based diabetes subgroups of adult-onset diabetes in the Japanese population. We obtained two major findings. First, we identified five diabetes subgroups in Japanese patients, similar to those in Caucasians [8,9,10,11]. However, there were a few critical distinctions in clinical features between Japanese and Caucasian people. Second, we found differences in the risk of diabetic complications among the subgroups as reported. However, our study considered modifiable risk factors (BMI, A1c, eGFR, smoking, hypertension, and dyslipidemia) as well as non-modifiable risk factors (age, sex, and diabetes duration) and therefore we obtained more clinically relevant results. As such, we clarified that the SIRD cluster was at increased risk of DKD, and the SAID subgroup, but not the SIDD subgroup, was at increased risk of diabetic retinopathy, after adjusting for modifiable risk factors. Interestingly, the SIRD cluster was not at increased risk of coronary artery disease after adjusting for modifiable risk factors. Our results demonstrate that clustering-based subgroups are applicable in estimating and monitoring diabetic complications in Japanese diabetic subjects.

### 4.1. Distribution and Clinical Features of Subgroups

By using the methods established by Ahlqvist et al. [8], we replicated the five exclusive subgroups of adult-onset diabetes in Japanese patients. The SAID subgroup was positive for islet-related autoantibodies and was young at onset. The SIDD subgroup had a severe insulin deficiency and the highest A1c. The SIRD subgroup was the highest in BMI, HOMA 2-IR, and HOMA2-B. The MOD subgroup had a higher BMI and was slightly younger than the MARD subgroup. These points were all similar to those in studies in Scandinavia [8], China, and the United States [9]. The reproducibility among different ethnic populations suggests that this method is applicable to Japanese populations similar to how they are to Caucasians [8,9,10,21]. However, there are differences, too. East Asians, including Japanese, have been reported to have a lower capacity for insulin secretion and lower insulin resistance compared to Caucasians [22,23]. This may at least partly explain the difference in the frequency of SAID, SIDD, and SIRD subgroups between Japanese and Caucasians. Second, HOMA2-IR and age at diagnosis in the SIRD subgroup were significantly lower than in Caucasians [8,9,10,11]. When compared between Caucasians and Chinese [9], there was the same trend for the SIRD cluster (Chinese was lower in HOMA2-IR and age). Combined, it might suggest that East Asians, with low endogenous capacity for insulin secretion, cannot compensate for increased insulin requirements with aging and thus develop diabetes at a younger age.

### 4.2. Association of Five Diabetic Subgroups with Diabetic Complications

#### 4.2.1. Diabetic Kidney Disease (DKD)

After correcting for the known risk factors (Model 2), we first demonstrated that SIRD is a strong independent risk factor for developing DKD, low eGFR, and proteinuria [24,25]. Dennis et al. reported that SIRD and MARD clusters were not at increased risk of developing DKD, when adjusted for baseline eGFR [21]. However, their observation period was short (5 years) for assessing DKD and did not consider other risk factors for DKD. Safai et al. [11] also reported that the odds ratio in DKD disappeared after adjustment for risk factors. However, their studies are cross-sectional. Our study was a long-term cohort study and considered additional modifiable risk factors, so that may cause the discrepancy with previous reports. Several possible mechanisms have been proposed for the association between insulin resistance and DKD [26,27]. Ahlqvist and colleagues [8] reported that participants with SIRD have a phenotype similar to that of participants with NAFLD because of the TM6SF2 genotype [28,29]. Zaharia et al. observed in their German cohort that the insulin resistance index in the glucose clamp technique, hepatic fat content, and progression of liver fibrosis were more prevalent characteristics in the SIRD subgroup than in the other subgroups [10]. The mechanism of CKD onset via hepatic insulin resistance has been reported in previous studies [30,31,32]. Since patients in the SIRD cluster are obese, this subgroup may develop CKD as obesity-related glomerulopathy [33] rather than classic diabetic nephropathy. In fact, our data showed that the prevalence of diabetic retinopathy was low (only 25.0% of SIRD patients with DKD) but that of NAFLD was high (66.7%). Combined, NAFLD-mediated development of CKD may be operative in SIRD.

#### 4.2.2. Diabetic Retinopathy

We found significant differences in the risk of diabetic complications among the subgroups. The SAID and SIDD subgroups were at a high risk of diabetic retinopathy when considering age, sex, and duration of diabetes (Model 3). Ahlqvist et al. reported that only the SIDD cluster was at risk for retinopathy in the Scania Diabetes Registry (SDR) cohort. The reason for the discrepancy between this study and the SDR cannot be determined from the current study. Since the risk of diabetic retinopathy in type 1 diabetes (SAID in the current study) is well known [34,35], the results of the current study are reasonable. When considered for A1c, the risk of SIDD, but not SAID, disappeared (Model 4). Collectively, it might be suggested that SAID itself, regardless of blood glucose control, is an independent risk factor for diabetic retinopathy due to unknown mechanism(s).

#### 4.2.3. Coronary Artery Disease

Ahlqvist et al. [8] and Dennis et al. [21] reported that the risk of coronary artery disease did not differ between the five clusters when adjusted for age and sex. In this study, we constructed two multivariate models and found that SIRD was a risk factor in Model 5 (adjusted for non-modifiable factors, such as age, sex, and diabetes duration), but not in Model 6 (adjusted for modifiable factors, such as BMI, smoking, hypertension, and dyslipidemia). Insulin resistance, the representative feature of SIRD, is a common pathological condition underlying diabetes, hypertension, and dyslipidemia in obese individuals and is strongly linked to the onset of coronary artery disease [36,37,38]. Our results support the notion that management of modifiable risk factors may be crucial for preventing coronary artery disease, even in diabetic patients with insulin resistance [39].

### 4.3. Study Limitations

Our study has limitations. First, because this was a retrospective study, the six variables used for clustering were not necessarily measured at onset, so they may have been affected by treatments such as diet, exercise, and medications. However, as the duration of diabetes was relatively short in almost all patients, the effect on clustering was not expected to be significant. Most patients (88.1%, 1339 of 1520) were checked for variables within 1 year (median 0, inter quarter range (0, 0)) years after diagnosis and the longest time for variables measured since diagnosis was eight years. The original study [8] suggested that the five clusters may be stable even as the disease duration progresses. Second, there may be a bias that patients at high risk of complications were recruited because the study was conducted at a single university hospital and its branch hospitals. Our patients may have poorer control of diabetes or have other risk factors for DKD such as hypertension and dyslipidemia, as compared to patients who visit a practitioner or a community hospital. Third, in our study, any patient who had diabetes with a low eGFR with or without proteinuria can be classified as DKD, but some cases of non-diabetic kidney disease were included incorrectly as DKD. Even in the Fukushima CKD cohort, renal biopsy was performed only in a small number of patients enrolled, and thus potential non-diabetic renal diseases cannot be excluded. In addition, because the diagnosis of complications depends on the ICD-10 code, some diabetic complications (e.g., peripheral artery disease: PAD≈) may not be accurately diagnosed. Finally, the participants in our study have a smaller database size than the Swedish study, so future confirmatory studies are required.

## 5. Conclusions

We identified five subgroups of adult-onset diabetes in the Japanese population and identified their risk of diabetic complications. Our results indicate that these classifications may be a helpful tool for predicting the risk of diabetic complications and for providing better intervention.

## Figures and Tables

**Table 1:** Baseline characteristics of clustering-based subgroups.

	Cluster 1: SAID	Cluster 2: SIDD	Cluster 3: SIRD	Cluster 4: MOD	Cluster 5: MARD	*p*-Value
*n* (%)	68 (5.4)	238 (19.0)	90 (7.2)	363 (28.9)	496 (39.5)	
Male, %	48.5	58.0	50.0	49.0	60.3	0.008
Age, Years	55 (41–62)	57 (49–65)	54 (41–64)	57 (49–65)	61 (53–68)	<0.001
Age at Diagnosis, Years	48 (35–56)	51 (42–58)	48 (39–57)	53 (45–60)	56 (47–62)	<0.001
Diabetes Duration, Years	5 (0–9)	3 (0–10)	2 (0–7)	1 (0–7)	3 (0–10)	0.005
BMI, kg/m^2^	23.1 (21.0–27.0)	24.7 (22.1–27.6)	28.3 (25.5–34.1)	26.1 (22.9–29.7)	24.0 (21.7–26.7)	<0.001
Systolic Blood Pressure, mmHg	133 (118–152)	132 (120–146)	137 (122–153)	133 (120–148)	133 (122–146)	0.630
Diastolic Blood Pressure, mmHg	80 (71–89)	76 (69–85)	79 (71–88)	78 (70–86)	77 (69–84)	0.066
Smoking, %	20.6	23.5	21.1	21.5	15.1	0.046
Family History of Diabetes, %	42.6	46.2	36.7	37.2	36.1	0.090
Plasma Glucose, mg/dL	193 (139–263)	237 (180–294)	135 (109–214)	136 (112–196)	142 (120–181)	<0.001
HbA1c, %	8.6 (7.3–9.7)	10.3 (9.3–11.8)	7.4 (6.5–8.7)	7.0 (6.4–8.3)	7.1 (6.5–7.7)	<0.001
HbA1c, mmol/mol	70.5 (56.3–82.5)	89.1 (78.1–105.7)	57.4 (47.5–71.6)	53.0 (46.4–67.2)	54.1 (47.5–60.6)	<0.001
HbA1c at the Follow-Up, %	7.7 (7.0–8.5)	7.4 (6.8–8.3)	6.8 (6.2–7.2)	6.7 (6.3–7.2)	6.9 (6.6–7.3)	<0.001
HbA1c at the Follow-Up, mmol/mol	60.4 (53.0–69.8)	57.6 (50.8–66.8)	50.3 (44.2–54.6)	49.7 (45.3–55.2)	51.9 (48.1–56.3)	<0.001
HOMA2-B	32.7 (12.2–65.2)	19.8 (18.0–47.6)	143.2 (130.1–164.6)	78.9 (69.8–94.4)	44.0 (29.6–53.8)	<0.001
HOMA2-IR	0.92 (0.58–2.20)	1.20 (0.72–2.07)	3.09 (2.19–3.86)	2.01 (1.41–2.68)	1.11 (0.66–1.70)	<0.001
eGFR, mL/min/1.73 m^2^	84 (67–103)	87 (69–103)	73 (52–88)	79 (64–90)	77 (64–91)	<0.001
Triglycerides, mg/dL	99 (62–161)	123 (84–172)	148 (97–207)	133 (97–183)	106 (79–160)	<0.001
LDL Cholesterol, mg/dL	112 (93–130)	118 (94–145)	123 (94–146)	117 (92–143)	117 (94–140)	0.422
Hypertension, %	63.2	70.6	84.4	76.9	77.2	0.008
Dyslipidemia, %	73.5	83.2	95.6	88.7	82.7	<0.001
CKD, %	14.7	13.4	34.4	19.6	20.6	0.001
Proteinuria, %	16.2	20.6	27.0	22.5	16.8	0.093
NAFLD, %	33.8	53.8	66.7	63.4	44.2	<0.001
Polyneuropathy, %	35.3	31.1	23.3	21.8	16.9	<0.001
Retinopathy, %	29.4	28.2	14.4	15.7	12.1	<0.001
Coronary artery disease, %	14.7	8.8	6.7	15.4	10.7	0.038
Stroke, %	4.4	5.5	6.7	4.4	6.0	0.824
Peripheral artery disease, %	4.4	2.9	0.0	3.6	3.4	0.461
Metformin, %	11.8	31.1	23.3	20.7	25.2	0.005
Insulin therapy, %	58.8	29.4	23.3	10.2	21.6	<0.001

Data are presented as median (25–75th percentile), or *n* (%). P values were obtained by Kruskal–Wallis test or Chi-square test. SAID: severe autoimmune diabetes; SIDD: severe insulin-deficient diabetes; SIRD: severe insulin-resistant diabetes; MOD: mild obesity-related diabetes; MARD: mild age-related diabetes; BMI: body mass index; HOMA2-B: homeostatic model assessment 2 estimates of β-cell function; HOMA2-IR: homeostatic model assessment 2 estimates of insulin resistance; HbA1c: hemoglobin A1c; eGFR: estimated glomerular filtration rate; LDL: low density lipoprotein; CKD: chronic kidney disease; NAFLD: nonalcoholic fatty liver disease.

**Table 2:** Cox regression analysis comparing risk of diabetic complications.

**Diabetic Kidney Disease**			**Model 1**		**Model 2**	
**Variables**	**Events (%)**	**Censored**	**HR (95% CI)**	***p*-Value**	**HR (95% CI)**	***p*-Value**
Cluster 1: SAID	23 (47.9)	25	1.23 (0.79–1.91)	0.361	1.08 (0.69–1.70)	0.742
Cluster 2: SIDD	70 (44.3)	88	1.04 (0.79–1.39)	0.773	0.86 (0.60–1.23)	0.404
Cluster 3: SIRD	28 (68.3)	13	2.38 (1.58–3.57)	<0.001	2.19 (1.44–3.34)	<0.001
Cluster 4: MOD	117 (51.1)	112	1.40 (1.10–1.79)	0.006	1.28 (0.99–1.64)	0.055
Cluster 5: MARD	156 (46.2)	182	1.00 (ref)		1.00 (ref)	
Age			1.04 (1.03–1.05)	<0.001	1.03 (1.02–1.04)	<0.001
Sex (Male)			1.05 (0.85–1.28)	0.667	1.02 (0.83–1.26)	0.841
Diabetes Duration			1.01 (0.99–1.02)	0.282	1.01 (0.99–1.02)	0.469
BMI					0.99 (0.98–1.02)	0.911
HbA1c (mmol/mol)					1.01 (0.99–1.01)	0.059
eGFR					0.98 (0.97–0.99)	<0.001
Smoking					1.13 (0.86–1.48)	0.381
Hypertension					1.06 (0.83–1.14)	0.658
Retinopathy					1.42 (1.09–1.87)	0.011
**Diabetic Retinopathy**			**Model 3**		**Model 4**	
**Variables**	**Events (%)**	**Censored**	**HR (95% CI)**	***p*-Value**	**HR (95% CI)**	***p*-Value**
Cluster 1: SAID	22 (45.8)	26	2.41 (1.50–3.86)	<0.001	1.81 (1.10–3.00)	0.020
Cluster 2: SIDD	64 (37.4)	107	1.81 (1.32–2.48)	<0.001	1.04 (0.69–1.55)	0.866
Cluster 3: SIRD	23 (29.9)	54	1.33 (0.84–2.10)	0.222	1.08 (0.66–1.75)	0.770
Cluster 4: MOD	64 (20.9)	242	0.98 (0.71–1.34)	0.877	0.89 (0.64–1.24)	0.490
Cluster 5: MARD	103 (23.6)	333	1.00 (ref)		1.00 (ref)	
Age			0.99 (0.98–1.00)	0.214	0.99 (0.98–1.00)	0.112
Sex (Male)			1.16 (0.91–1.48)	0.233	1.11 (0.86–1.43)	0.433
Diabetes Duration			1.04 (1.03–1.06)	<0.001	1.04 (1.03–1.06)	<0.001
BMI					0.99 (0.97–1.02)	0.477
HbA1c (mmol/mol)					1.02 (1.01–1.02)	<0.001
Smoking					1.19 (0.87–1.62)	0.277
Hypertension					1.01 (0.76–1.34)	0.948
Dyslipidemia					1.02 (0.73–1.42)	0.911
CKD					1.36 (1.00–1.86)	0.050
**Coronary Artery Disease**			**Model 5**		**Model 6**	
**Variables**	**Events (%)**	**Censored**	**HR (95% CI)**	***p*-Value**	**HR (95% CI)**	***p*-Value**
Cluster 1: SAID	5 (8.6)	53	0.79 (0.32–1.96)	0.607	0.80 (0.43–3.04)	0.643
Cluster 2: SIDD	43 (19.8)	174	1.49 (1.02–2.17)	0.041	1.30 (0.78–2.18)	0.313
Cluster 3: SIRD	19 (22.6)	65	1.94 (1.17–3.22)	0.011	1.39 (0.81–2.38)	0.233
Cluster 4: MOD	46 (15.0)	261	1.15 (0.79–1.66)	0.464	1.04 (0.71–1.52)	0.841
Cluster 5: MARD	76 (17.2)	367	1.00 (ref)		1.00 (ref)	
Age			1.04 (1.02–1.05)	<0.001	1.03 (1.02–1.05)	<0.001
Sex (Male)			1.60 (1.18–2.17)	0.002	1.31 (0.90–1.90)	0.162
Diabetes Duration			1.02 (0.99–1.03)	0.070	1.02 (1.00–1.04)	0.047
BMI					1.01 (0.98–1.04)	0.725
HbA1c (mmol/mol)					1.01 (0.99–1.01)	0.296
Smoking					1.49 (1.05–2.13)	0.027
Hypertension					2.68 (1.66–4.35)	<0.001
Dyslipidemia					2.03 (1.21–3.42)	0.008
CKD					1.38 (0.98–1.94)	0.064

Diabetic kidney disease was defined as eGFR < 60 mL/min/1.73 m^2^ or proteinuria. SAID: severe autoimmune diabetes; SIDD: severe insulin-deficient diabetes; SIRD: severe insulin-resistant diabetes; MOD: mild obesity-related diabetes; MARD: mild age-related diabetes; BMI: body mass index; HbA1c: hemoglobin A1c; eGFR: estimated glomerular filtration rate; CKD: chronic kidney disease.

## Supplementary Materials

The following are available online at https://www.mdpi.com/2077-0383/9/7/2083/s1, Figure S1: Enrollment flow chart, Table S1: Multiple comparison of baseline characteristics between five subgroups, Table S2: Multiple log-rank test for a pair of five subgroups in Kaplan–Meier survival analysis (Figure 2).
