# Supplementary material for: Factors Associated with Risk of Diabetic Complications in Novel Cluster-Based Diabetes Subgroups: A Japanese Retrospective Cohort Study
Source: J Clin Med. 2020 Jul 2;9(7):2083. doi: 10.3390/jcm9072083 (PMC7408659; doi:10.3390/jcm9072083)
Supplement: Supplementary file 1 [file jcm-09-02083-s001.pdf]

Supplementary Materials

Table S1. Multiple comparison of baseline characteristics between five subgroups.

	Over all	SAID vs SIDD	SAID vs SIRD	SAID vs MOD	SAID vs MARD	SIDD vs SIRD	SIDD vs MOD	SIDD vs MARD	SIRD vs MOD	SIRD vs MARD	MOD vs MARD
Sex (Male), %	0.008	0.999	0.999	0.999	0.690	0.999	0.350	0.999	0.999	0.690	0.013
Age, years	<0.001	0.176	0.974	0.117	<0.001	0.452	0.999	<0.001	0.402	<0.001	<0.001
Age at diagnosis, years	<0.001	0.117	0.646	0.003	<0.001	0.831	0.235	<0.001	0.119	<0.001	0.006
Diabetes duration, years	0.005	0.981	0.358	0.041	0.738	0.548	0.015	0.884	0.965	0.866	0.062
BMI, kg/m^2	<0.001	0.415	<0.001	0.003	0.942	<0.001	0.004	0.181	<0.001	<0.001	<0.001
Systolic blood pressure, mmHg	0.630	0.999	0.879	0.999	0.999	0.536	0.964	0.956	0.774	0.671	0.999
Diastolic blood pressure, mmHg	0.066	0.257	0.996	0.665	0.201	0.348	0.836	0.999	0.813	0.253	0.620
Smoking, %	0.046	0.999	0.999	0.999	0.999	0.999	0.999	0.073	0.999	0.999	0.184
Family history of diabetes, %	0.090	0.999	0.999	0.999	0.999	0.999	0.310	0.110	0.999	0.999	0.999
Plasma glucose, mg/dL	<0.001	0.016	0.009	<0.001	<0.001	<0.001	<0.001	<0.001	0.999	0.985	0.931
HbA1c, %	<0.001	<0.001	0.047	<0.001	<0.001	<0.001	<0.001	<0.001	0.211	0.027	0.884
HbA1c, mmol/mol	<0.001	<0.001	0.047	<0.001	<0.001	<0.001	<0.001	<0.001	0.207	0.026	0.883
HbA1c at the follow-up, %	<0.001	0.682	<0.001	<0.001	<0.001	<0.001	<0.001	<0.001	0.999	0.073	<0.001
HbA1c at the follow-up, mmol/mol	<0.001	0.682	<0.001	<0.001	<0.001	<0.001	<0.001	<0.001	0.999	0.073	<0.001
HOMA2-B	<0.001	0.989	<0.001	<0.001	0.191	<0.001	<0.001	<0.001	<0.001	<0.001	<0.001

HOMA2-IR	<0.001	0.720	<0.001	<0.001	0.999	<0.001	<0.001	0.082	<0.001	<0.001	<0.001
eGFR, mL/min/1.73 m^2	<0.001	0.990	0.028	0.084	0.075	<0.001	<0.001	<0.001	0.424	0.385	0.999
Triglycerides, mg/dL	<0.001	0.155	0.003	<0.001	0.624	0.089	0.085	0.347	0.844	0.001	<0.001
LDL choresterol, mg/dL	0.422	0.507	0.463	0.628	0.795	0.998	0.985	0.822	0.962	0.850	0.980
Hypertension, %	0.008	0.630	0.040	0.180	0.140	0.140	0.520	0.380	0.620	0.620	0.970
Dyslipidemia, %	<0.001	0.349	0.002	0.015	0.349	0.042	0.349	0.941	0.349	0.024	0.107
Chronic kidney disease, %	0.001	0.999	0.062	0.999	0.999	<0.001	0.333	0.152	0.047	0.047	0.999
Proteinuria, %	0.093	0.999	0.999	0.999	0.999	0.999	0.999	0.999	0.999	0.330	0.410
NAFLD, %	<0.001	0.034	<0.001	<0.001	0.276	0.143	0.096	0.09	0.644	<0.001	<0.001
Polyneuropathy, %	<0.001	0.999	0.706	0.173	0.005	0.765	0.107	<0.001	0.999	0.765	0.541
Retinopathy, %	<0.001	0.999	0.181	0.079	0.002	0.089	0.003	<0.001	0.999	0.999	0.621
Coronary artery disease, %	0.038	0.999	0.999	0.999	0.999	0.999	0.250	0.999	0.420	0.999	0.420
Stroke, %	0.824	0.999	0.999	0.999	0.999	0.999	0.999	0.999	0.999	0.999	0.999
Peripheral artery disease, %	0.461	0.999	0.999	0.999	0.999	0.999	0.999	0.999	0.999	0.999	0.999
Metformin, %	0.005	0.025	0.689	0.689	0.174	0.689	0.046	0.689	0.999	0.999	0.689
Insulin therapy, %	<0.001	<0.001	<0.001	<0.001	<0.001	0.675	<0.001	0.077	0.006	0.816	<0.001

Overall P values were obtained by Kruskal-Wallis test and Chi-square test between five subgroups.P values by Holm test for a pair of five subgroups were shown.BMI: body mass index; HOMA2-B: homeostatic model assessment 2 estimates of β-cell function; HOMA2-IR: homeostatic model assessment 2 estimates of insulin resistance; HbA1c: Hemoglobin A1c; eGFR: estimated glomerular filtration rate; LDL: low density lipoprotein; NAFLD: nonalcoholic fatty liver disease.

Table S2. Multiple log-rank test for a pair of five subgroups in Kaplan-Meier survival anaysis (Figure 2).

	**Over all**	**SAID vs SIDD**	**SAID vs SIRD**	**SAID vs MOD**	**SAID vs MARD**	**SIDD vs SIRD**	**SIDD vs MOD**	**SIDD vs MARD**	**SIRD vs MOD**	**SIRD vs MARD**	**MOD vs MARD**
Diabetic kidney disease	0.025	0.865	0.080	0.575	0.981	0.019	0.142	0.680	0.142	0.032	0.246
Chronic kidney disease	0.094	0.820	0.330	0.660	0.820	0.170	0.210	0.820	0.510	0.170	0.210
Proteinuria	0.005	0.528	0.018	0.381	0.699	0.018	0.668	0.528	0.036	0.003	0.241
End-stage kidney disease	0.092	0.730	0.730	0.910	0.930	0.910	0.260	0.260	0.260	0.260	0.910
Diabetic retinopathy	<0.001	0.292	0.105	<0.001	<0.001	0.292	<0.001	<0.001	0.191	0.272	0.626
Coronary artery disease	0.133	0.280	0.280	0.310	0.280	0.560	0.280	0.340	0.280	0.280	0.640

P values by log-rank test for overall five subgroups or P values by multiple rog-rank test for a pair of five subgroups.

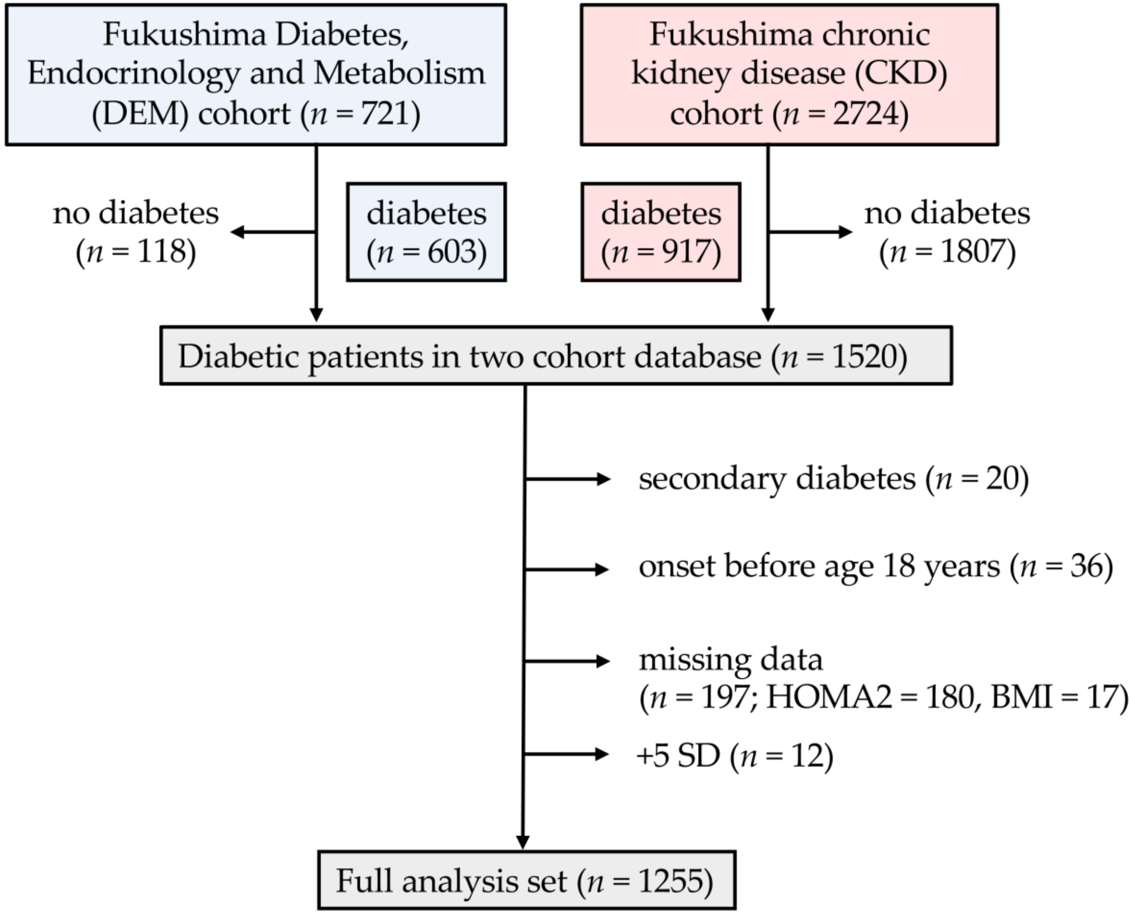

Figure S1. Enrollment flow chart Among the 3444 patients surveyed, 1520 were diagnosed as having diabetes. Patients with secondary diabetes, onset before age 18 years, missing data, and extreme outliers (> 5 SDs from the mean) were excluded. CKD: chronic kidney disease; HOMA: homeostatic model assessment; BMI: body mass index; SD: standard deviation
